# Special Notice

**Published:** 1890-06

**Authors:** F. E. Daniel

**Affiliations:** Secretary, Austin, Texas


### SPECIAL NOTICE.

By resolution adopted at the Fort Worth meeting of the Texas State Medical Association, it was made the duty of the Secretary to secure the name, postoffice address, aud date of death of every member of the Texas State Medical Association who has died since organization to date, together with the year when admitted to membership; and to publish the same in the forthcoming Transactions. (At the same meeting a resolution was adopted setting aside an hour on the first day of each annual meeting for memorial services in honor of the dead members; hence the necessity of a correct list.)

This is a herculean task imposed on the Secretary, and he calls on the members to help him; especially the older members who have data as to members deceased before his connection with the Association. Any one therefore knowing the facts in regard to any one or more of the deceased members, will confer a favor on the Secretary and on the Association by communicating them to the Secretary at once.

F. E. DANIEL, Secretary,
Austin, Texas.

In this connection it is but just and right to remind members that a resolution was also adopted authorizing the Secretary to send the Transactions only to those who have paid their dues. Should any fail to get this handsome volume (with portrait of President elect,) they will know the reason why; and must not think hard of their clever and obliging Secretary for obeying orders. Remit to him, or to Treasurer J. Larendon, Houston.

